# From the Heart to the Gut: A Case Report of a Rare Complication After Coronary Artery Bypass Graft Surgery

**DOI:** 10.7759/cureus.46372

**Published:** 2023-10-02

**Authors:** Reynat Jimenez-Hernandez, Juan Vazquez-Fuster, Patricia Rivera

**Affiliations:** 1 Internal Medicine, VA Caribbean Healthcare System, San Juan, PRI; 2 Cardiology, VA Caribbean Healthcare System, San Juan, PRI

**Keywords:** acute dilated colon, transverse colon, colonoscopic decomression, coronary artery bypass grafting(cabg), ogilvie's syndrome

## Abstract

Coronary artery bypass graft (CABG) surgery has a major role in the management of obstructive coronary artery disease, especially in patients with diabetes or multiple vessel disease. Currently, in the USA, the annual incidence rate of CABG has been reported to be approximately 400,000. Overall, gastrointestinal (GI) complications occur in less than 2% of patients undergoing open-heart surgery. Acute colonic pseudo-obstruction, also known as Ogilvie's syndrome, is a disorder characterized by dilatation of the colon in the absence of an anatomic lesion that obstructs the flow of intestinal contents. This condition occurs in 0.06% of patients following cardiac surgery, and in CABG patients, the reported incidence is approximately 0.046%. In this report, we discuss a case of a patient who developed Ogilvie’s syndrome after undergoing CABG.

## Introduction

Open-heart surgeries are complex procedures that carry high risks and are associated with a broad range of potential postoperative complications. Reported gastrointestinal (GI) complications among patients undergoing cardiac surgical procedures include bleeding, mesenteric ischemia, pancreatitis, cholecystitis, and ileus. It is estimated that approximately less than 2% of patients may develop GI complications [1-2]. Currently, in the USA, the incidence rate of coronary artery bypass graft (CABG) surgery cases is approximately 400,000 annually [3,4]. Despite these procedures being very common, there is limited data regarding GI complications associated with them. Acute colonic pseudo-obstruction, also known as Ogilvie's syndrome, is a disorder characterized by dilatation of the colon in the absence of an anatomic lesion that obstructs the flow of intestinal contents [1,5-8]. It is a rare condition and has been identified in only a few patients after CABG.

## Case presentation

**Figure 1:**
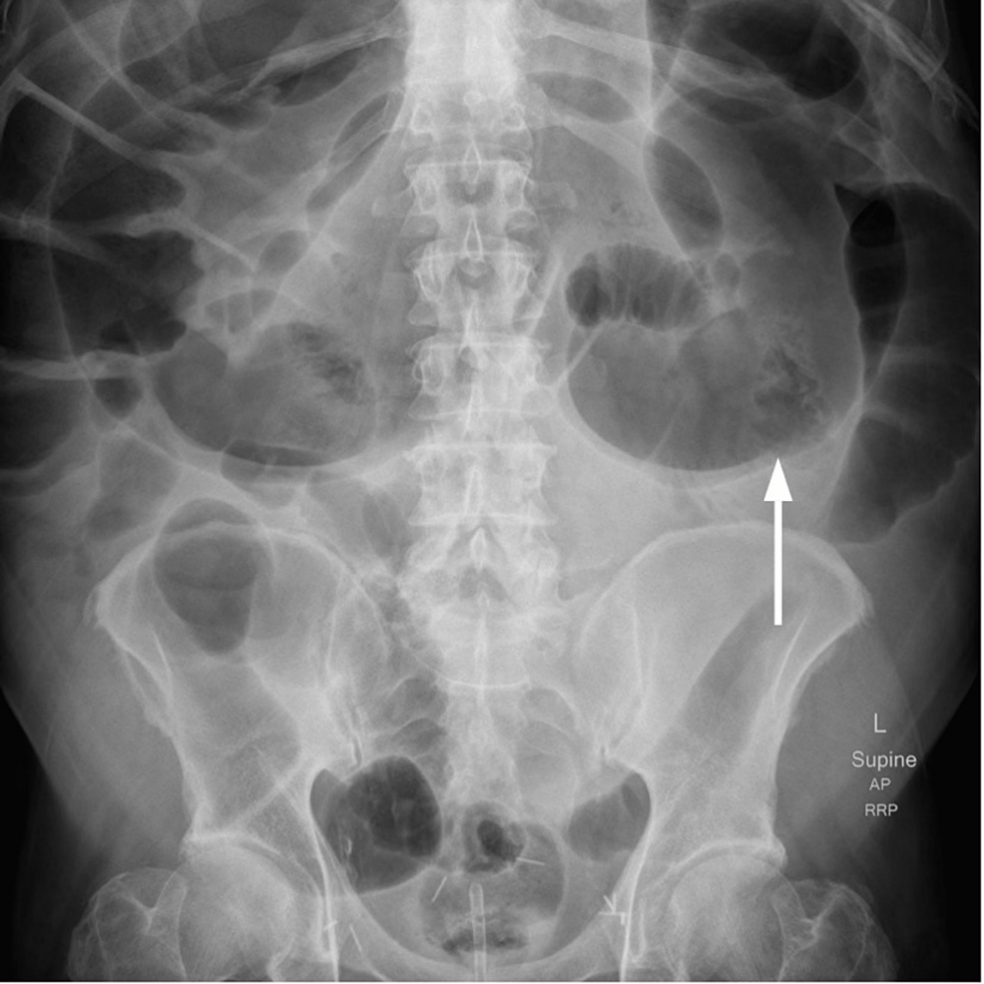
Abdominal X-ray The arrow demonstrates colon distention

**Figure 2:**
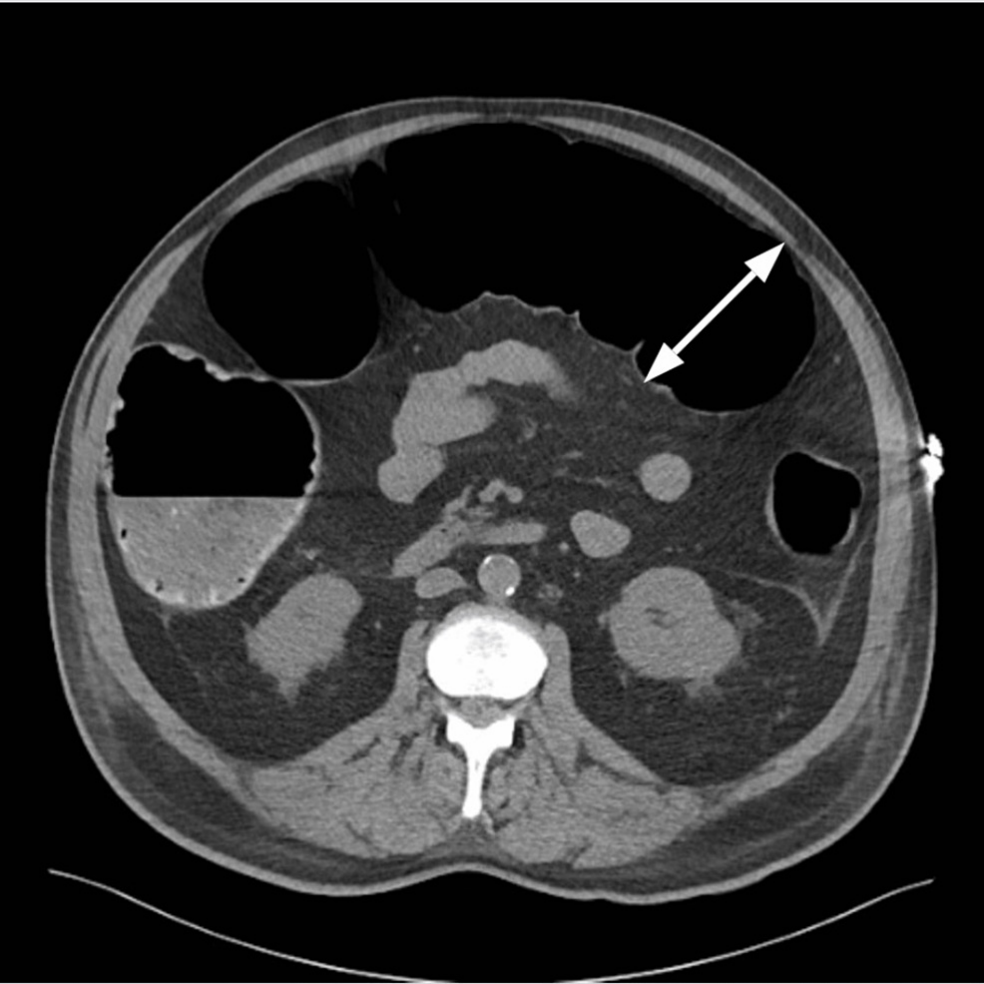
Abdominal CT - transverse view The arrow demonstrates transverse colon distention of around 11 cm CT: computed tomography

**Figure 3:**
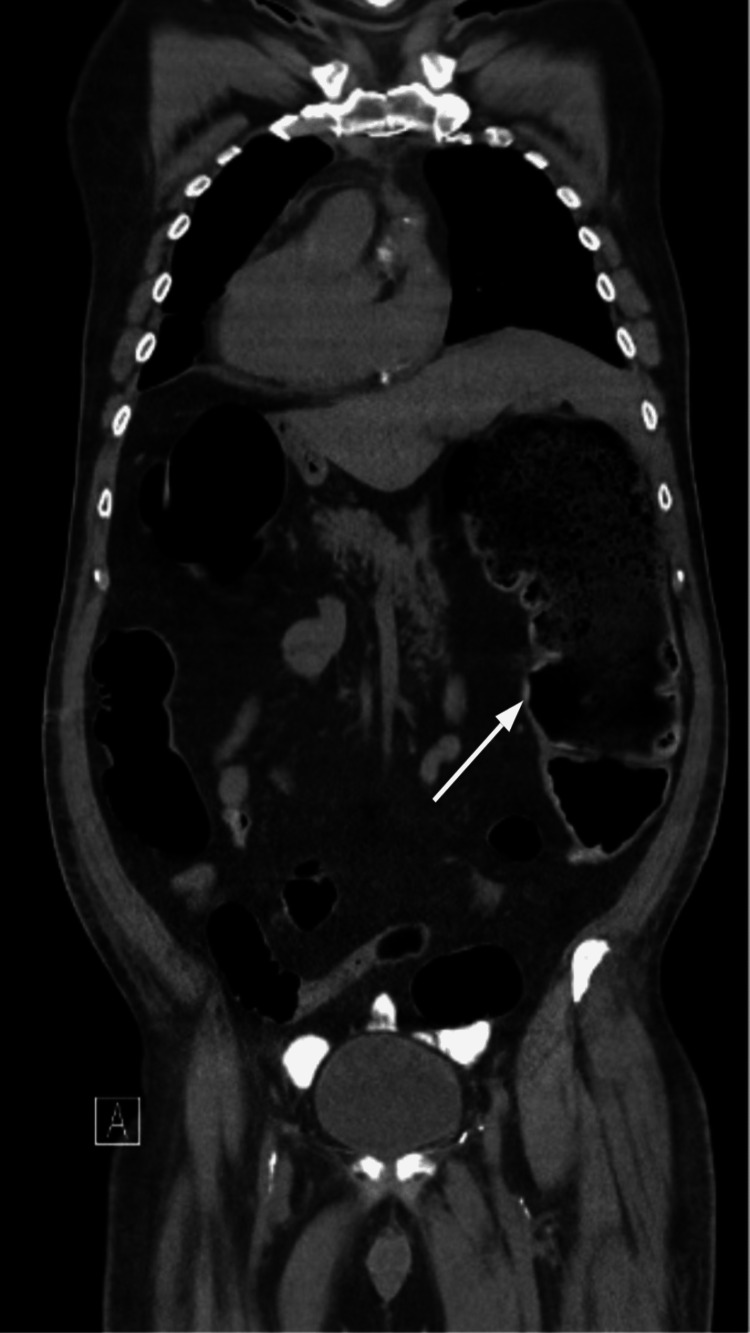
Abdominal CT - coronal view The arrow demonstrates cecum distention of around 9 cm CT: computed tomography

A 65-year-old male, a former smoker, with hypertension, type two diabetes mellitus, and dyslipidemia presented with symptoms of angina. Cardiac catheterization revealed triple vessel vessel coronary artery disease and he was scheduled to undergo CABG. After the surgery, the patient was transferred to the ICU for postoperative management. On postoperative day (POD) four, the patient experienced nausea and abdominal pain. Physical exam was remarkable for distended abdomen, hypoactive bowel sounds, and tympanitic sound to percussion. Despite the abdominal complaint, the patient was alert, oriented, and hemodynamically stable. He tolerated a liquid diet and passed flatus, but there were no bowel movements. Abdominal ultrasound was negative for intra-abdominal pathology, and blood tests did not reveal any abnormal electrolyte imbalance. Abdominal X-ray revealed dilation of the central small bowel as well as the colon (Figure 1). Subsequent abdominal CT (Figures 2, 3) showed distention of the colon with the cecum and transverse colon measuring 9 cm and 11 cm respectively without any evidence of an obstruction, findings consistent with Ogilvie’s syndrome. The patient was initially managed with bowel rest, nasogastric tube for intermittent suction, and rectal tube without any improvement. Due to the persistence of symptoms, he underwent colonoscopic decompression on POD day nine, which led to the complete resolution of symptoms.

## Discussion

Acute colonic pseudo-obstruction or Ogilvie's syndrome is defined as colonic dilation in the absence of obstruction. While its pathophysiology has not been fully established, it is associated with bowel parasympathetic dysfunction. It has a higher prevalence in male patients over the age of 60 years [7,8]. It has been linked with orthopedic surgery, obstetric/gynecological procedures, neurological diseases, malignancy, infection, metabolic imbalances, and medications that affect bowel motility. Ogilvie's syndrome occurs in approximately 0.06% of cardiac surgery patients, and, more specifically, in 0.046% of CABG surgery patients [1,7].

Ogilvie's syndrome should be suspected in patients with abdominal distention, altered bowel habits, abdominal pain, nausea, or vomiting who present with acute illness or have undergone recent surgery. Imaging modalities such as abdominal radiography, CT, or water-soluble contrast enema are needed to establish the diagnosis [1]. The dilation of the colon greater than 8-12 cm places patients at high risk for perforation. Initial treatment includes conservative management with nasogastric suction, rectal tube for decompression, and bowel rest. In cases where symptoms persist, neostigmine or colonoscopic decompression should be considered. Colonoscopic or surgical decompression is recommended when conservative measurements fail or if the patient is at a very high risk for perforation. If not managed in a prompt manner, significant dilation may lead to colon perforation and peritonitis [6,9].

Our patient had colonic pseudo-obstruction after undergoing CABG. Imaging showed colon dilation up to 11 cm at the transverse colon (Figure 2). Conservative management with bowel rest, nasogastric suction, and rectal tube failed to show any improvement. To prevent perforation, the patient underwent a colonoscopy, and colon decompression was achieved without any additional complications. The exact pathophysiology of Ogilvie’s syndrome is currently unknown. Prompt identification of symptoms and timely evaluation are keys to adequate management and favorable outcomes in these patients.

## Conclusions

Ogilvie's syndrome is a rare postoperative complication, and very few cases of it have been identified following CABG surgery. Close clinical monitoring is critical during the postoperative period. Physicians need to maintain close monitoring of the patient's vital signs as well as lab and physical exam results. Early identification of symptoms and acute management can help avoid fatal complications and achieve favorable outcomes.
